# Intersecting stigma, social exclusion and coping mechanisms among women living with obstetric fistula in rural northern Ghana: a qualitative study

**DOI:** 10.3389/frph.2026.1923194

**Published:** 2026-08-21

**Authors:** Nawara Sulemana Abilla, Ernestina Asiedua, Joyce B. P. Pwavra, Salamatu Issah, Ephraim Senkyire

**Affiliations:** 1 Department of Nursing, College of Nursing and Midwifery, Ntotroso, Ghana; 2 Department of Maternal and Child Health, School of Nursing and Midwifery, University of Ghana, Legon-Accra, Ghana; 3 Department of Midwifery, Nursing and Midwifery Training College- Kpembe, Salaga- Savannah Region, Ghana; 4 Department of Nursing & Midwifery, Ga West Municipal Hospital, Ghana Health Service, Amasaman-Accra, Ghana

**Keywords:** coping strategies, Ghana, obstetric fistula, qualitative research, reintegration, reproductive health, social exclusion, stigma

## Abstract

**Background:**

Obstetric fistula is a preventable childbirth injury that remains socially devastating for women in low-resource settings. Existing evidence on obstetric fistula stigma is largely derived from treatment centres and urban settings, leaving limited understanding of the lived experiences of women in underserved rural communities. This study addresses this knowledge gap by qualitatively examining stigma, coping strategies, and support needs among obstetric fistula survivors in Kpandai District, northern Ghana.

**Methods:**

A qualitative exploratory descriptive design was used. Fourteen women aged 30–70 years who were living with obstetric fistula were recruited through purposive and snowball sampling until data saturation was reached. Semi-structured face-to-face interviews were audio-recorded, transcribed verbatim, and analysed using Braun and Clarke's thematic analysis approach. Link and Phelan's stigma framework informed the analytic interpretation.

**Results:**

Five themes were generated: labelling and stereotypes, separation from community, status loss and discrimination, coping mechanisms, and recommendations and support needs. Participants described name-calling, association of fistula with sin or curses, abandonment by relatives, exclusion from social and religious gatherings, and loss of social respect. Coping strategies included faith and spirituality, concealment of leakage and odour, withdrawal from public spaces, and limited psychosocial support from relatives or friends. Participants prioritised accessible fistula treatment, community education, livelihood support, and social inclusion.

**Conclusion:**

Obstetric fistula-related stigma extends beyond the physical injury, profoundly affecting women's social identity, dignity, and wellbeing. Comprehensive survivor-centred care that integrates surgical repair with psychosocial support, community stigma reduction, livelihood restoration, and strengthened referral systems is essential to promote successful reintegration and improve long-term health and social outcomes.

## 1 Introduction

Obstetric fistula remains one of the most devastating yet preventable childbirth injuries. It continues to represent a major public health and human rights challenge in many low- and middle-income countries (1). It is primarily caused by prolonged obstructed labour without timely access to emergency obstetric care, resulting in abnormal communication between the birth canal and the urinary bladder (vesicovaginal fistula), the rectum (rectovaginal fistula), or both. Consequently, affected women experience continuous leakage of urine, faeces, or both through the vagina (2–4). Although obstetric fistula has been virtually eliminated in high-income countries through timely access to quality intrapartum care and emergency obstetric services, it remains prevalent in resource-constrained settings where poverty, child marriage, prolonged home labour, inadequate transportation, weak referral systems, shortages of skilled birth attendants, and limited access to comprehensive emergency obstetric and newborn care persist (3, 5–7). Consequently, obstetric fistula continues to reflect broader inequities in maternal healthcare despite being almost entirely preventable and treatable when timely obstetric interventions are available (6, 8).

The global burden of obstetric fistula remains substantial. Approximately two million women are estimated to be living with untreated obstetric fistula worldwide, while between 50,000 and 100,000 new cases occur annually (9). Sub-Saharan Africa bears a disproportionate share of this burden, accounting for an estimated 30,000–130,000 new cases each year, with a prevalence of approximately 10.9 cases per 1,000 live births (5, 7). Ghana also continues to experience a considerable burden, with an estimated incidence of 1.8 cases per 1,000 deliveries, corresponding to approximately 1,352 women affected annually among 751,205 deliveries (10). Despite ongoing national and international initiatives aimed at eliminating obstetric fistula, the persistence of new cases indicates continuing deficiencies in access to timely, high-quality maternal healthcare and equitable health services (11, 12).

The consequences of obstetric fistula extend well beyond the physical injury itself (13, 14). Persistent urinary and faecal incontinence often results in chronic skin excoriation, recurrent infections, offensive odour, pain, infertility, sexual dysfunction, reduced mobility, and impaired ability to perform household, occupational, and caregiving responsibilities (2, 3). However, the social consequences are frequently more profound than the physical symptoms. Women living with obstetric fistula often experience rejection, marital disruption, economic deprivation, social isolation, psychological distress, and diminished quality of life (15–17). In many communities, the continuous leakage and associated odour are interpreted as signs of uncleanliness, spiritual punishment, witchcraft, or moral failure, leading affected women to be blamed for their condition rather than recognised as survivors of preventable childbirth complications (18). Such experiences frequently prevent women from participating in family life, community gatherings, religious activities, and income-generating work, thereby reinforcing cycles of poverty, dependence, and social exclusion (13, 14).

Stigma has increasingly been recognised as one of the most debilitating consequences of obstetric fistula because it shapes women's lived experiences long after the physical injury has occurred (19, 20). Beyond individual prejudice, stigma functions as a social process that influences identity, relationships, access to resources, and healthcare utilisation. Women who experience stigma are less likely to disclose their condition, seek timely treatment, or successfully reintegrate into their communities after surgical repair. Consequently, understanding stigma is essential because eliminating the fistula alone does not necessarily restore social acceptance, dignity, or quality of life (21, 22). Addressing stigma has therefore become an important component of comprehensive fistula care and aligns with current global recommendations advocating holistic approaches that combine surgical treatment with psychosocial rehabilitation and community reintegration (12).

Link and Phelan's conceptualisation of stigma provides a useful theoretical lens for understanding these experiences. The framework defines stigma as the convergence of labelling, stereotyping, separation, status loss, and discrimination operating within social, cultural, and institutional power structures (23). This framework is particularly relevant to obstetric fistula because visible symptoms such as continuous leakage and offensive odour make the condition socially recognisable, allowing negative labels and stereotypes to emerge. These processes may subsequently lead to social exclusion, diminished social status, internalised shame, economic marginalisation, and delayed healthcare seeking. Applying this framework enables a deeper understanding of how stigma extends beyond individual experiences to reflect broader social and structural inequalities that shape women's lives.

Existing evidence from Ghana and other sub-Saharan African countries has documented the psychological distress, social isolation, marital disruption, barriers to care, and reintegration challenges experienced by women living with obstetric fistula (10, 15–17). Previous studies have also highlighted that stigma influences healthcare utilisation and long-term wellbeing (20, 24). However, much of the available evidence has been generated from treatment centres, urban populations, or broader regional studies, with relatively limited attention to the unique experiences of women residing in rural districts of northern Ghana. Rural communities often experience greater poverty, poor transport infrastructure, long distances to specialist services, lower educational attainment, and stronger traditional beliefs regarding illness causation, all of which may intensify stigma and delay access to care (11, 21). Kpandai District represents one such underserved setting where women face substantial barriers to maternal healthcare, yet little is known about how stigma is experienced, negotiated, and managed within this sociocultural context.

A qualitative approach was therefore considered most appropriate because stigma is a socially constructed and deeply personal phenomenon that cannot be adequately understood through quantitative measures alone (25, 26). Qualitative inquiry allows women to describe, in their own words, how obstetric fistula has influenced their identities, relationships, community participation, coping strategies, and healthcare experiences (27). It also facilitates exploration of culturally embedded meanings, survivor perspectives, and support needs that are often overlooked by epidemiological studies (28). Such evidence is critical for informing survivor-centred interventions that address not only clinical treatment but also psychosocial recovery, community reintegration, and stigma reduction (29).

Given these evidence gaps, this study explored the stigma experiences and coping strategies of women living with obstetric fistula in Kpandai District in the Northern Region of Ghana. Guided by Link and Phelan's stigma framework, the study specifically examined experiences of labelling and stereotyping, separation from community, status loss and discrimination, and the coping strategies adopted by affected women. It also explored survivor-identified support needs to inform comprehensive fistula care, community reintegration, and stigma reduction interventions within rural Ghanaian settings.

## 2 Methods

### 2.1 Study design

A qualitative exploratory descriptive design was used to explore the lived stigma experiences and coping mechanisms of women living with obstetric fistula. This design was appropriate because the study sought to generate in-depth descriptions of participants' meanings, emotions, social relationships, and everyday responses to stigma rather than quantify associations (30, 31).

### 2.2 Study settings

The study was conducted at Kpandai District in the Northern Region of Ghana. The district is predominantly rural and is home to diverse ethnic groups, including Konkomba, Basaari, Chokosi, Kotokoli, and others. Livelihoods largely depend on farming and trade. The setting is marked by limited access to specialist health services, transport constraints, and strong community and cultural structures that shape health beliefs and social responses to reproductive morbidity.

### 2.3 Participants and recruitment

Participants were women aged 30–70 years living with obstetric fistula in Kpandai District who were willing to take part in an individual interview. Eleven women who met the inclusion criteria were purposively recruited directly from the community with the help of the community health nurse. Participants were given information about the nature of the research. After interviewing each newly recruited participant, the snowball technique was employed. They were asked whether they knew other eligible women who would be interested in the study. This snowballing approach led to the recruitment of three other women, bringing the total sample size to fourteen participants. Women who were critically ill or unwilling to participate voluntarily were excluded. Recruitment continued until data saturation, the point at which additional interviews no longer produced new themes or analytic insights (32).

### 2.4 Data collection

Data were collected through semi-structured, face-to-face interviews using an interview guide (IG) (see Supplementary File S1) developed by the first author from the study objectives and the Link and Phelan stigma framework. The IG was prepared through extensive literature review and in consultation with research committee members. The guide explored name-calling and stereotypes, experiences of avoidance or exclusion, perceived loss of social standing, discrimination in family or community life, coping strategies, community support, and desired changes in the health system or community. The interviews were conducted in English, Twi, and Konkomba (local dialect) by the first author because these are the languages that both the researcher and participants understand and can speak fluently. The interview started with the establishment of rapport between the researcher and the participants. This was followed by a thorough explanation of the purpose and processes of the study. Participants were assured of voluntary participation and anonymity. They were also told that after giving consent, they could opt out at any point in the study. The main questions were asked, followed by probes. At the end of each interview, there was a closure where participants could also ask for clarifications. The interview was conducted at places that the participants deemed convenient. Some were conducted in participants' homes, and for those who found their homes not convenient for the interviews, a place was arranged for them within the hospital setting.

Interviews were audio-recorded with participants' consent and lasted approximately 45–60 min. In addition, more flexible open-ended questions and intentional silence during interviews were encouraged to allow participants to freely express their thoughts. Participants were encouraged to always express themselves freely, and each was allowed to reflect on his or her experiences. During the interview, participants' responses were probed or redirected as needed to ensure that adequate and detailed responses were elicited from them. The researcher also took field notes of all nonverbal communications to improve understanding of the data and aid in analysis.

Three pretest interviews were conducted in Wulensi in Nanumba South District, a nearby setting with similar population characteristics, to refine the interview guide and assess interview duration. Audio recordings were transcribed verbatim and anonymised using participant identification codes. The researcher kept the interview materials in his possession with a password for safety and to protect the participants' confidentiality. Respondent demographic data were separated from interview data, and “care was taken” to ensure that no linkages occur between them. Participants were given false names with which they were identified to reduce the risk of associations being made with them.

### 2.5 Data analysis

Data analysis was conducted concurrently with data collection using an iterative, inductive thematic analysis approach (33). Interviews were transcribed verbatim, back translated into English by two independent language experts and repeatedly reviewed to ensure familiarity with the data. Meaningful units were coded using NVivo 12, with similar codes grouped into sub-themes and subsequently synthesised into broader themes. As analysis progressed, new themes were continuously incorporated and refined by examining their coherence across the entire dataset and their relevance to the coded extracts. The final themes were systematically defined and interpreted to construct a logical narrative addressing the research questions, supported by illustrative participant quotations guided by Link and Phelan's framework (23). EA and JP verified the data. Initially, 5 participants were interviewed, and initial analysis of the data was performed by 3 Authors: EA, JBPP and NSA. The findings were compared, and consensus was reached through discussion, guided by the *a priori* themes.

### 2.6 Trustworthiness

Trustworthiness was enhanced through prolonged engagement with the data, verbatim transcription, use of illustrative quotations, maintenance of an audit trail, and reflexive attention to the researcher's role in interpretation. Credibility was supported by careful rapport-building before interviews and by allowing participants adequate time to narrate their experiences. Again, credibility was enhanced using probing questions during interviews. The initial interviews were scrutinised by the supervisors and also presented to colleagues for feedback. In addition, the investigator conferred with her superiors to guarantee that the responses furnished by the participants are precisely recorded in the results. Moreover, six participants ‘ transcripts were reviewed and validated by them. Dependability and confirmability were supported through documentation of decisions made during coding and theme development. Transferability was strengthened by providing contextual details about the study setting and participant characteristics. A detailed audit trail of the whole data gathering experience was maintained; this was to enable replication of the study by another researcher.

### 2.7 Ethical considerations

Ethical approval was obtained from the Institutional Review Board of the Noguchi Memorial Institute for Medical Research, University of Ghana (approval number: IRB 00001276). Administrative permission was obtained from the Kpandai District Health Directorate. Written informed consent was obtained from all participants before interviews. Participants were informed that participation was voluntary and that they could withdraw at any time without penalty. Identifying information was removed from transcripts, and participants were represented with codes. Because interviews could evoke distressing experiences, psychological support was arranged for participants if needed.

### 2.8 Reflexivity

All authors had professional backgrounds in maternal health, with clinical, teaching, and research experience in maternal and reproductive healthcare. While this disciplinary expertise facilitated an informed understanding of obstetric fistula and its broader health and social implications, it also required continuous self-reflexivity throughout the research process. We recognised that our professional knowledge, prior assumptions, clinical experiences, and commitment to improving maternal health could influence how data were collected, interpreted, and represented.

To enhance reflexivity, the primary researcher maintained a reflexive journal throughout the study to document personal reflections, assumptions, emotional responses, and emerging interpretations during data collection and analysis. This ongoing process encouraged critical examination of how the researchers' perspectives might shape interactions with participants, coding decisions, theme development, and interpretation of the findings. Reflexive journaling also promoted conscious bracketing of preconceived ideas, thereby reducing subjectivity and enhancing the credibility and transparency of the analytical process.

To further minimise individual bias, coding and interpretation were discussed among the research team through regular peer debriefing and collaborative coding. These discussions challenged assumptions, explored alternative interpretations, and ensured that the final themes remained grounded in participants' narratives rather than the researchers' expectations. Throughout the analysis, priority was given to preserving participants' voices by using verbatim quotations and ensuring that interpretations accurately reflected their lived experiences.

We acknowledge that complete objectivity is neither possible nor expected in qualitative research. Consequently, the findings should be interpreted within the context of the researchers' perspectives as maternal health professionals. Nevertheless, the deliberate use of reflexive journaling, peer coding, collaborative interpretation, and continuous critical reflection strengthened the trustworthiness, credibility, and confirmability of the study by ensuring that the analysis remained closely connected to participants' accounts while recognising the influence of the researchers' positionality.

## 3 Results

Fourteen women participated in the study. Participants were aged 30–70 years and had lived with fistula for 4–34 years. Most participants were married at the time of interview, although two were widowed and two were divorced. The onset of fistula was associated with major livelihood changes, with several participants moving from trading into farming or no occupation. See Table 1 summarises the participant characteristics.

**Table 1:** Summary of participant characteristics.

Participant ID	Age (years)	No. of Children	Years with fistula	ANC Visits	Marital Status	Previous Occupation	Current Occupation	Religion	Ethnicity
P1	40	5	4	4	Married	Trading	None	Muslim	English
P2	32	5	5	3	Married	Trading	None	Muslim	Konkomba
P3	40	6	4	3	Married	Trading	Farming	Muslim	Konkomba
P4	40	5	8	3	Divorced	Trading	Farming	Muslim	Twi
P5	40	5	8	5	Married	Trading	Farming	Christian	Konkomba
P6	31	3	5	2	Divorced	Trading	None	Traditionalist	English
P7	30	5	5	2	Married	Trading	None	Muslim	Twi
P8	50	0	12	0	Divorced	Farming	Farming	Christian	Konkomba
P9	70	1	34	0	Widow	Farming	None	Traditionalist	Twi
P10	42	7	10	0	Married	Trading	None	Traditionalist	Konkomba
P11	41	4	10	0	Co-habiting	Trading	Farming	Christian	Konkomba
P12	40	5	9	1	Co-habiting	Farming	Farming	Christian	Konkomba
P13	65	1	20	1	Widow	Trading	Farming	Muslim	Konkomba
P14	40	4	6	4	Divorced	Trading	Farming	Muslim	Twi

Thematic analysis generated five themes and sixteen subthemes. The analysis was both inductive and theory-informed. Link and Phelan's stigma framework guided the interpretation of stigma-related findings, while allowing new concepts to emerge from participants' narratives. Consistent with the framework, the first three themes represent the core stigma process. Because participants' experiences of labelling and stereotyping were closely intertwined, these two components were combined into a single analytical theme: **Labelling and stereotypes**. The remaining framework components are reflected in the themes **Separation from community** and **Status loss and discrimination**, the latter combining the closely related processes of status loss and discriminatory experiences described by participants. Two additional inductively derived themes, **Coping mechanisms** and **Recommendations and support needs,** captured experiences beyond the stigma framework and reflected participants' responses to stigma and their priorities for improving fistula care and reintegration. The relationship between the analytical themes and Link and Phelan's framework is presented in Table 2.

**Table 2:** Themes, subthemes, and alignment with link and Phelan's stigma framework.

Theme	Subthemes	Interpretive focus	Alignment with Link & Phelan framework (2001)
Labelling and stereotypes	Verbal insults and name-calling; association with sin and curses; loss of dignity and identity	How women were socially marked and reduced to the fistula condition	Labelling and stereotyping
Separation from community	Abandonment by family; social withdrawal; neglect by community members	How stigma created physical, social, and emotional distance	Separation
Status loss and discrimination	Loss of social respect; avoidance and rejection by others	How women lost social standing, voice, and ordinary participation	Status loss and discrimination
Coping mechanisms	Faith and spirituality; concealment strategies; withdrawal and avoidance; psychosocial support	How women managed leakage, shame, rejection, and emotional distress	Emergent theme (outside framework)
Recommendations and support needs	Healthcare access; community education and advocacy; livelihood empowerment; social inclusion	Survivor-identified priorities for treatment, reintegration, and dignity restoration	Emergent theme (outside framework)

### 3.1 Labelling and stereotypes

Labelling and stereotypes were central to participants' experiences. The women reported verbal insults and name-calling, society attributing their condition to sin and curses, and loss of dignity and identity.

#### 3.1.1 Verbal insults and name-calling

Participants reported direct insults, whispering, mockery, and public ridicule. Such name-calling made usual social interactions painful and encouraged avoidance of public spaces.

“They talk bad about me and call me names like smelly woman, leaking woman and useless woman. Hmm.. madam, even when I pass by, they whisper and laugh. **(P3, 40 years, living with fistula for 4 years)”**

#### 3.1.2 Association with sin and curses

Several participants described community explanations that framed fistula as divine punishment, a curse, or a consequence of immoral conduct. These beliefs shifted attention away from preventable childbirth injury and deepened blame.

“Some of the elders in my village told me that my sickness came from the gods because I offended them. Most of the opinion leaders even think that I have gotten the sickness as a punishment for the sins I have committed. **(P6, 31 years, living with fistula for 5 years).**

“People in our community often say hurtful things about women like me who have obstetric fistula. They think we're cursed or that we've done something terribly wrong to deserve this condition. Some believe it's a punishment from the gods for past sins or that we're just unlucky. It's hard to hear these things because it adds to the pain and isolation we [women with fistula] already feel” **(P9, 70-year-old woman, living with fistula for 34 years)**

#### 3.1.3 Loss of dignity and identity

Repeated labelling undermined women's sense of personhood. Participants described feeling stripped of dignity and recognized only through their condition.

“I have lost my dignity… sometimes I wonder if I will ever be accepted again. The sickness has taken away my pride as a woman; I cannot satisfy my husband or have another child. I am not a real woman anymore.” **(P1, 40-year-old woman, living with fistula for 4 years).** “People's words make me doubt myself. I used to be confident, but now I feel small and useless. I no longer feel like a human being. The urine keeps coming out, and I smell it all the time. I cannot even sit among people. I feel useless” **(P2, 32 years old woman, living with fistula).**

Women described being known by the odor and leakage and not their names, and public meanings attached to fistula also led to the women being blamed and labelled. Community members used humiliating terms that made them feel visible as “different” and undeserving of the least respect. This loss of identity was particularly painful because social respect in the community was closely tied to being a wife, mother, trader, and active participant in communal life.

### 3.2 Separation from community

The stigma experienced by the women translated into separation from family and community. This was evidenced through abandonment by family, social withdrawal and neglect by community members.

#### 3.2.1 Abandonment by family

Participants described abandonment by spouses, relatives, and household members. This rejection left women with limited emotional and financial support, particularly where they could no longer work as traders or farmers.

“Everyone left me. My friends, my husband, even my own nieces do not come close to me. My husband does not want to see me. If I use his things like a cup or a bowl, he will not use them again.” **(P10, 42 years, living with fistula for 10 years)**

“Yes, before I got this condition, I used to cook for my family and take care of my husband. Now, they don't let me cook, and my husband has abandoned me for another woman” **(P12, 40 years old woman, living with fistula for 12 years)**

#### 3.2.2 Social withdrawal

Fear of humiliation led some participants to stop attending markets, mosques, churches, weddings, village meetings, and other social spaces.

“I no longer go to social gatherings. I prefer to stay alone. I have stopped going to the mosque and other social gatherings. I do not want to embarrass myself or make people uncomfortable.” **(P8, 50 years, living with fistula for 5 years)**

#### 3.2.3 Neglect by community members

Participants also described the silence of community leaders and other influential people. In the absence of active advocacy, women experienced neglect as another form of social abandonment.

“…No, they rarely intervene. Most leaders seem to ignore the issue or pretend it does not exist.” People who used to greet me with respect now avoid eye contact. I feel invisible in my own village.” **(P4, 40-year-old woman, living with fistula for 8 years).** “An imam once spoke about compassion for women like me during a sermon, but it didn't lead to any real change. Before this problem, I joined women's meetings and funerals. Now, they do not even tell me when there is a gathering. They say I bring shame.” **(P5, 40year-old woman, living with fistula for 8years**)

Community silence or neglect reinforced women's invisibility and reduced opportunities for support. Some women were physically abandoned, while others withdrew voluntarily to avoid ridicule. Some women relocated to places far from their homes. The withdrawal protected them from direct ridicule but also intensified loneliness*.*

### 3.3 Status loss and discrimination

Participants described a sharp loss of social standing after developing fistula. Women who had once been respected as wives, mothers, advisers, and economically active community members became targets of avoidance and pity. The condition altered how others greeted them, sat with them, invited them, or listened to them.

#### 3.3.1 Loss of social respect

Participants reported that the respect they previously received declined after the onset of fistula. Social values attached to marriage, motherhood, neatness, and productivity were disrupted by leakage and odor.

“Before the sickness, people respected me; I could work, go to gatherings, and be with others. I was the leader of a dance group in my community. In fact, a lot of people used to come to me for advice. But now, people look down on me; they do not even greet me. **(P6, 31 years, living with fistula for 5 years).** “I was once called Madam in my community because I was hardworking and neat. Now I have lost all the respect I once had.” **(P9, 70 years, living with fistula for 34 years).**

#### 3.3.2 Avoidance and rejection by others

Avoidance occurred in ordinary spaces such as meetings, gatherings, and conversations. Some community members behaved as though fistula was contagious. Some women abandoned the roles they played in society because either society did not allow them to continue in their roles or they themselves could not continue in the roles due to the continuous leakage.

“Whenever I sit somewhere, people move away. Some cover their noses; others just leave without saying anything. So how could I have continued as the women's leader? I quit, but no one even followed up to ask me why I quit. It makes me feel like I do not belong.” (P4, 40 years, living with fistula for 8 years). In the village meetings, no one wants to sit near me. They say that I smell bad and should stay at home. **(P1, 40 years, living with fistula for 4 years).**

Status loss was evident where husbands brought new wives to the house or stopped eating foods prepared by the wives who suffered obstetric fistula. This loss of status did not necessarily have to be announced; it could be subtle. If a fistula prevented a woman from playing social roles she used to play, then she automatically lost her status either as a wife or a community or church leader. This reinforced the idea that women living with fistula did not belong in shared social spaces anymore.

### 3.4 Coping mechanisms

Despite persistent stigma, participants developed coping strategies that allowed them to endure daily life. These strategies reflected resilience but also revealed the lack of sufficient formal support. The coping mechanisms included: faith and spirituality, concealment strategies, avoidance and psychosocial support. Coping was therefore both adaptive and constrained.

#### 3.4.1 Faith and spirituality

Faith was the most visible coping strategy. Participants used prayer, religious belief, and trust in divine healing to endure abandonment and uncertainty.

“I do not have anyone to assist me. I only rely on God as my source of hope. I believe only God can heal me. When everyone rejects me, I pray day and night, and that gives me hope to live.” **(P14, 40 years, living with fistula for 6 years)**. “Sometimes, I want to give up, but when I pray, I get peace in my heart. I know my God has not forgotten me.” **(P12, 40 years, living with fistula for 9 years)**.

#### 3.4.2 Concealment strategies

Participants tried to conceal leakage and odor through frequent bathing, perfumes, disposable pads, cloths, and multiple layers of clothing. These strategies allowed limited participation in social life but were restricted by poverty and the recurring cost of supplies.

“I use Pampers and perfume whenever I can afford. Because I cannot work like I used to, sometimes I cannot afford to buy Pampers[diapers], so I always put many pieces of cloth between my legs so that people will not notice the wetness or smell.” **(P2, 32 years, living with fistula for 5 years**). “Sometimes I use old clothes since I cannot buy Pampers all the time. I also bathe several times a day and change my clothes often so that people will not know I am leaking” **P12, 40 years, living with fistula for 9 years)**.

#### 3.4.3 Withdrawal and avoidance

Withdrawal was both a coping strategy and a consequence of stigma. By avoiding crowds, women reduced public exposure to ridicule. However, isolation also limited social support and worsened loneliness.

“I just try to avoid crowds. I spend most of my time in the room avoiding negative labels. I prefer to stay alone in my room. When I go out, people make faces because of the smell, so it is better I keep to myself.” **(P9, 70 years, living with fistula for 34 years).**

#### 3.4.4 Psychosocial support

Some participants had limited support from friends or relatives. Such support did not remove the condition but helped women feel seen, encouraged, and less lonely to a lesser extent.

“One of my friends always visits me and prays with me. Even when others avoided me, she stayed by my side. That makes me feel I am not alone.” **(P5, 40 years, living with fistula for 8 years**). “My sister never left me. She encourages me every day and tells me I will get well. Her love gives me strength to continue living”. **(P2, 32 years, living with fistula for 5 years)**.

The findings revealed that coping among women with obstetric fistula is a multi-layered process characterised by spirituality, personal resilience and sustained efforts to deal with and navigate persistent stigma. Participants adopted various strategies to maintain their personal dignity and psychological wellbeing. Support from family and friends played a major role in helping the women cope better with the condition. Spirituality had a profound effect on the women's ability to cope, gave meaning to suffering and helped them resist despair.

### 3.5 Recommendations and support needs

Participants were not passive recipients of suffering. They articulated practical recommendations for health care, community education, livelihoods, and inclusion. These survivor-defined priorities point to the need for integrated fistula programming.

#### 3.5.1 Healthcare access

Women prioritized nearby, affordable, and competent fistula treatment. Delayed access to surgical repair and post-operative care prolonged suffering and reinforced stigma.

“They should get us a hospital that can cure that sickness here. If we have those people here, I do not think this sickness would have taken so long like this. **(P2, 32 years, living with fistula for 5 years**)”. “If the government can help us get free medical care, it will reduce our suffering. (P6, 31 years, living with fistula for 5 years)”

#### 3.5.2 Community education and advocacy

Participants emphasized community education to correct beliefs that fistula is caused by sin or curses. They viewed nurses, radio programs, community leaders, and survivor testimonies as important channels for stigma reduction.

“When the nurses came to educate our community, people started to understand that fistula is not a curse. Since then, they treat me better now; more community engagement will help”. **(P4, 40 years, living with fistula for 8 years)**. “If people understand this condition is medical, they will not call us names and shun our company”. **(P11, 40 years, living with fistula for 4 years)**.

#### 3.5.3 Livelihood empowerment and social inclusion

Loss of income deepened dependency and vulnerability. Participants recommended vocational training and income-generating support after healing. They also desired acceptance and participation in family, religious, and community life.

“When we are healed, then they [health workers] should at least get us trained in some activities like bread making and sewing to enable us to survive”. **(P7, 30 years, living with fistula for 5 years).** “This condition does not define me. After all, I was not born with it. I deserve kindness and inclusion”. **(P13, 65 years, living with fistula for 20 years).**

“For a long time, I stayed away from meetings because I thought people would reject me. But after the nurses came to educate the community, the women's group encouraged me to join them again.” **(P3, 42-year-old woman, living with fistula for 5 years).**

Women in this study intimated that creating awareness on the disease condition will reduce abandonment by family and community, thereby reducing the stigma. Access to health care and livelihood empowerment were also mentioned as a means of empowering the women and ensuring social inclusion.

## 4 Discussion

This study demonstrates that obstetric fistula extends beyond a physical childbirth injury to become a socially constructed and stigmatised identity that shapes women's interactions, self-perceptions, and opportunities for social participation. The findings are well explained by Link and Phelan's stigma framework, which conceptualises stigma as a dynamic process involving labelling, stereotyping, separation, status loss, and discrimination (23). Rather than occurring as isolated experiences, these interconnected processes reinforce one another and perpetuate long-term social exclusion. By illustrating how stigma is embedded within the socio-cultural and economic realities of a rural Ghanaian setting, the study adds contextual evidence to the growing literature from Ghana and sub-Saharan Africa, highlighting that the consequences of obstetric fistula are sustained not only by the physical condition itself but also by persistent structural barriers, cultural beliefs, and limited access to specialised care (34–37).

The findings indicate that stigma among women living with obstetric fistula begins with labelling and stereotyping, whereby participants were repeatedly referred to by derogatory names, associated with curses, sin, and immoral behaviour, and gradually came to define themselves through these negative social identities. Rather than being recognised as women living with a preventable childbirth injury, participants described becoming known primarily by their leakage, odour, and the meanings attached to these symptoms. Interpreted through Link and Phelan's framework, these experiences illustrate how labelling and stereotyping function as the entry point of the stigma process, transforming a treatable obstetric condition into a socially discrediting identity. Similar findings have been reported in Ghana and other sub-Saharan African settings, where obstetric fistula is commonly interpreted through spiritual, supernatural, or moral explanations rather than prolonged obstructed labour and health-system delays (16, 18, 38, 39). These beliefs reinforce blame, legitimise discriminatory attitudes, discourage disclosure, and contribute to delayed treatment seeking. The findings therefore highlight the need for culturally responsive health education that engages community beliefs while reframing obstetric fistula as a preventable and treatable childbirth complication.

The findings further demonstrate that stigma extended beyond negative labelling to social separation from family and community. Participants described abandonment by spouses and relatives, withdrawal from social and religious activities, and limited support from community leaders, suggesting that stigma disrupted their participation in everyday community life. Although some women intentionally withdrew to avoid ridicule, this strategy also reduced opportunities for social support and reinforced loneliness. These findings support Link and Phelan's proposition that labelling and stereotyping ultimately lead to social separation and exclusion. Similar experiences have been documented in Tanzania, Malawi, Ethiopia, and Ghana, where women with obstetric fistula frequently avoid markets, churches, mosques, and community gatherings because of anticipated embarrassment associated with leakage and odour (17, 18, 20, 24). In rural communities, where social identity is closely connected to family relationships and communal participation, prolonged separation may have lasting consequences for emotional wellbeing, social reintegration, and recovery. These findings suggest that successful fistula care should extend beyond surgical treatment to include family engagement, community support, and structured reintegration programmes.

The findings also reveal that obstetric fistula resulted in substantial status loss and discrimination, with participants describing the loss of respect, leadership roles, marital relationships, and valued social identities following the onset of the condition. Women who were previously recognised as wives, mothers, traders, advisers, and community leaders reported becoming objects of avoidance and pity, while some internalised these experiences and questioned their own worth. These findings illustrate how stigma is reinforced through prevailing gender norms that associate women's social value with marriage, motherhood, productivity, and bodily cleanliness. Similar findings have been reported across sub-Saharan Africa, where obstetric fistula has been shown to disrupt marital relationships, fertility expectations, economic participation, and social recognition (15, 19, 40). The findings further suggest that discrimination becomes internalised over time, as persistent social rejection gradually shapes women's self-perceptions and psychological wellbeing. Consequently, interventions should address both enacted stigma within communities and internalised stigma among survivors by integrating psychosocial support, community stigma reduction, and long-term social reintegration into comprehensive fistula care.

Women's coping strategies reflected adaptation within constrained social and economic circumstances rather than resolution of the underlying problem. Consistent with previous research, faith and spirituality functioned as important sources of hope, resilience, and emotional endurance during prolonged illness (38, 41). However, reliance on spiritual coping should not obscure the responsibility of health systems to provide timely, accessible, and comprehensive care. Similarly, practical strategies used to conceal urine leakage and odour demonstrate how women negotiated social participation despite persistent symptoms. The dependence of these strategies on personal financial resources illustrates how poverty and stigma intersect to intensify disadvantage, with limited economic capacity reducing women's ability to manage visible symptoms and increasing vulnerability to social exclusion. Comparable coping mechanisms have been described in Ethiopia and Ghana, where concealment practices represent adaptive responses to stigma rather than effective long-term solutions (40, 42). These findings underscore that coping should not be interpreted as evidence of adequate support but rather as an indication of unmet psychosocial and socioeconomic needs.

The findings emphasise that reducing fistula-related stigma requires interventions operating beyond the individual level. Surgical repair remains fundamental to treatment but is unlikely to achieve full recovery in the absence of supportive family, community, and health-system environments. Evidence from reintegration programmes indicates that combining surgical care with psychosocial counselling, livelihood restoration, family engagement, and community education facilitates social recovery and improves long-term wellbeing (19, 21, 22). Integrating these services within Ghana's district health system, Community-based Health Planning and Services (CHPS) programme, and existing social protection initiatives could provide more holistic and sustainable support for survivors. Furthermore, community education delivered through nurses, community health workers, local radio, and trusted traditional and religious leaders may help challenge misconceptions by reframing obstetric fistula as a preventable and treatable obstetric complication rather than a consequence of moral or spiritual failure. Such approaches are more likely to achieve sustained stigma reduction when they engage communities respectfully rather than confronting local beliefs in ways that may increase resistance (10, 12, 15).

The study also highlights important implications for policy and health systems. Improving access to specialist fistula services requires coordinated investments in timely referral pathways, transport support, psychosocial care, rehabilitation, and livelihood restoration to address the multidimensional consequences of the condition. The observed disruptions in employment and inactive health insurance status illustrate how financial insecurity and administrative barriers can delay treatment and perpetuate social exclusion. Consequently, strengthening national fistula strategies, improving surveillance and data systems, and integrating comprehensive post-repair support into routine maternal health services would contribute not only to successful surgical outcomes but also to sustainable social reintegration. Such policies recognise that successful fistula management should be evaluated not solely by fistula closure but also by women's ability to regain dignity, participate fully in community life, and achieve long-term psychosocial wellbeing. For future research, a longitudinal study could be conducted to explore how women with obstetric fistula experience stigma, social reintegration and coping evolve over time.

## 5 Limitations

This study was conducted in one rural district and involved fourteen women, so findings should be interpreted as context-specific rather than statistically generalizable. The study relied on participants' retrospective accounts, which may be affected by recall and emotional distress. Women who were more hidden, severely ill, or unwilling to disclose fistula may not have been reached. Nevertheless, the qualitative design generated detailed accounts of stigma, coping, and support needs that are analytically transferable to similar rural settings. The limitation of the snowballing approach was that participants could refer women with similar characteristics to themselves, and this could affect diversity and variation. The use of the community health nurse within the community in the recruitment of the participants for this study might have influenced their willingness to disclose negative or sensitive information. Lastly, the study relied on self-reported narratives, which may be influenced by recall bias or social desirability, particularly when discussing socially sensitive issues such as rejection and coping behaviours.

## 6 Conclusion

Obstetric fistula is not only a preventable childbirth injury but also a profoundly stigmatising condition that undermines women's dignity, social identity, and quality of life. Guided by Link and Phelan's stigma framework, this study demonstrates that stigma is experienced through interconnected processes of labelling, stereotyping, social exclusion, status loss, and discrimination. At the same time, women's coping strategies largely reflect adaptation to constrained social and economic circumstances rather than resolution of their needs. These findings underscore that successful fistula management requires more than surgical repair alone. Comprehensive survivor-centred care should integrate psychosocial support, community stigma reduction, livelihood restoration, and culturally responsive health education within routine maternal health services. Strengthening referral systems, expanding access to specialist fistula care, and implementing policies that support long-term social reintegration are essential to restoring not only women's physical health but also their dignity, social participation, and overall wellbeing.

## Data Availability

The raw data supporting the conclusions of this article will be made available by the authors, without undue reservation.
