# Supplementary material for: Intersecting stigma, social exclusion and coping mechanisms among women living with obstetric fistula in rural northern Ghana: a qualitative study
Source: Front Reprod Health. 2026 Aug 21;8:1923194. doi: 10.3389/frph.2026.1923194 (PMC13541671; doi:10.3389/frph.2026.1923194)
Supplement: Supplementary file 1 [file Table1.docx]

Supplementary Material

# 1 DATA COLLECTION INSTRUMENTS: INTERVIEW GUIDE

Code Number…………………

SECTION A: SOCIO-DEMOGRAPHIC DATA

a) Age (years)

b) Residential place (Village Name) ……………………………………………………

c) Ethnicity……………………………………………………………………………………

d) Religion……………………………………………………………………………………

e) Educational status………………………………………………………………………

f) Previous Occupation ………………………………………………………………………

g) Current Occupation……………………………………………………………………….

h) Languages Spoken ………………………………………………………………………

i) Marital status……………………………………………………………………………

j) Number of Children………………………………………………………………………

k) Duration of Labour……………………………………………………………………….

l) Place of labour…………………………………………………………………………….

m) Number of ANC Visits……………………………………………………………………

n) Years living with obstetric fistula…………………………………………………………

o) Number of months after repair……………………………………………………………

p) Conception after repair……………………………………………………………………

q) National health insurance: Active/ Not Active

SECTION B: The role of labeling and stereotypes associated with women with obstetric

fistulas.

1. What do people say about women with obstetric Fistula?

2. How does the name-calling affect women with obstetric fistulas?

3. Do you think the name-calling have affected how people treat you personally? In what ways?

4. How have this name calling impacted your sense of identity?

SECTION C: The experiences of separation of women with obstetric fistulas within their

communities.

5. How do people in your community usually react when they find out a woman has an obstetric

fistula?

6. Have you ever been avoided, rejected, or excluded from community activities, social

gatherings, or events?

7. Are there places or events in your community where you feel unwelcome or excluded because

of your condition?

8. Have any community leaders or religious figures addressed your condition or intervened in

your experience of separation?

SECTION D: The status loss and discrimination associated with living with obstetric fistulas.

9. Since developing obstetric fistula, have you noticed any changes in the way people treat you in

your community?

10. How do they treat you compared to before you had the condition?

11. Have you experienced any form of discrimination (e.g., in terms of work, family life, or

community participation) because of your condition?

12. How do you think the community views women with obstetric fistulas?

13. Do you feel that having obstetric fistula has affected your social status or standing in the

community?

If yes, how?

SECTION E: the coping mechanisms of women living with obstetric fistula.

14. In your opinion, how do women with obstetric fistula deal with the stigma associated with

their condition?

15. What are the strategies that helped you to deal with the stigma?

16. Are there any community-based organizational efforts to help these women and combat

stigma? If so, what are they?

17. How do you keep a positive outlook despite the difficulties you face?

18. What kind of support do you think would help women like you living with obstetric fistula in

dealing with the stigma?

19. Are there any changes you would like to see in the community or healthcare system to better

support women living with obstetric fistulas?

20. Is there anything else you would like to share about your experiences that we have not

discussed?

Thank you very much for your time and for sharing your experiences.that all information is clearly presented, the
